# Female genital mutilation and cutting: a survey of child abuse pediatricians

**DOI:** 10.1186/s12905-024-03119-7

**Published:** 2024-06-17

**Authors:** Grace Pipes, Spencer Dunleavy, Jocelyn Brown

**Affiliations:** 1 Columbia University Vagelos College of Physicians and Surgeons, New York, NY USA; 2 Cedars-Sinai Medical Center, 8700 Beverly Blvd, Los Angeles, CA USA; 3 Department of Family Medicine and Community Health, University of Pennsylvania, Philadelphia, PA USA; 4 Department of Pediatrics, Columbia University Vagelos College of Physicians and Surgeons, New York, NY USA

**Keywords:** Female Genital Mutilation/Cutting, Immigrant Health, Child Abuse Pediatrics, Knowledge and Attitudes, Medical Education

## Abstract

**Background:**

As global immigration from countries with a high prevalence of female genital mutilation and cutting (FGM/C) has grown in the United States (US), there is need for pediatricians to have adequate training to care for these patients. The objective of this study is to determine the level of knowledge and attitudes of child abuse pediatricians (CAPs) towards FGM/C in the US.

**Methods:**

This cross-sectional study distributed a peer-reviewed survey to US CAPs—members of the Helfer Society—to assess their attitudes, knowledge, clinical practice, and education about FGM/C. Data was analyzed using descriptive statistics, Kruskal–Wallis tests, and Fisher’s exact test.

**Results:**

Most of the 65 respondents were aware that FGM/C is illegal (92%) and agreed that it violated human rights (99%). Individuals reporting previous training related to FGM/C were significantly more likely to correctly identify World Health Organization types of FGM/C (*p* < 0.05) and report confidence in doing so (*p* < 0.05). Only 21% of respondents felt comfortable discussing FGM/C with parents from countries with a high prevalence of FGM/C. Sixty-three percent were not aware of the federal law, and 74% were not aware of their own state’s laws about FGM/C.

**Conclusions:**

US CAPs have high rates of training related to FGM/C; however, they need additional training to increase confidence and ability to identify FGM/C. FGM/C remains a topic that CAPs find difficult to discuss with families. With culturally sensitive training, CAPs have the opportunity to help manage and prevent the practice by serving as educators and experts for general pediatricians.

**Supplementary Information:**

The online version contains supplementary material available at 10.1186/s12905-024-03119-7.

## Background

**Fig. 1:**
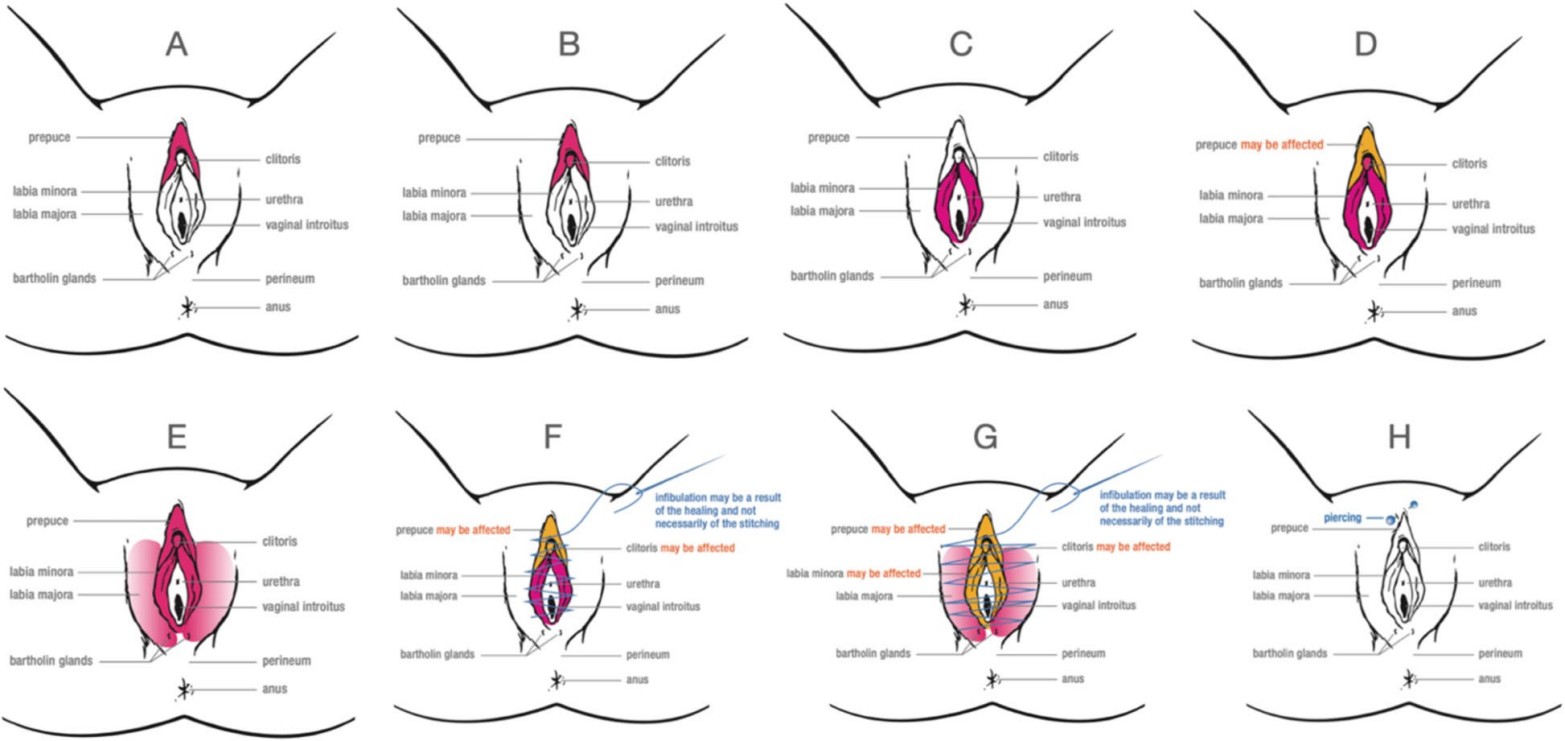
FGM/C WHO types and subtypes. Pink indicates areas affected; Orange indicates possible areas affected. Type I, clitoridectomy: (**A**) Type Ia, (**B**) Type Ib. Type II, excision: (**C**) Type IIa, (**D**) Type IIb, (**E**) Type IIc. Type III, infibulation: (**F**) Type IIIa, (**G**) Type IIIb. Type IV includes all other harmful procedures: (**H**) Type IV. Illustrations adapted with permission from the WHO [2]

Female genital mutilation/cutting (FGM/C) is defined by the World Health Organization (WHO) as the partial or total removal of the external female genitalia or other injury to the female genital organs for non-medical reasons [1]. FGM/C is a violation of human rights because it interferes with healthy genital tissue without any medical necessity or health benefit and can result in severe short- and long-term mental and physical health consequences [1, 2]. Despite this, more than 200 million girls and women alive today, in over 30 countries, have experienced FGM/C. Typically, FGM/C is performed on girls between the ages of 0 and 15 years, and the practice ranges from a prick of the genitals to complete infibulation (Fig. 1) [3, 4]. FGM/C is a deeply ingrained practice in a range of cultures and ethnicities. Though there is a misconception that religion requires FGM/C, it predates the Islamic and Christian religions, and mention of the practice is absent from both the Quran and the Bible [4]. Explanations for the practice include safeguarding virginity, aesthetics, prevention of rape, and ensuring fidelity. The practice establishes ethnic identity and ensures social acceptance, family honor, and marriageability [1].

Global immigration has resulted in significant growth of immigrant populations in the United States (US), including those from countries with a high prevalence of FGM/C [3, 5–7]. Therefore, it is likely that physicians will encounter more women who have received the procedure and more families that may consider it for their daughters. It is estimated that approximately 545,000 girls and women living in the US are at risk or have been cut, with approximately 200,000 being those under 18 years of age [6]. In the US, the practice of FGM/C was deemed illegal by the Federal Prohibition of Female Genital Mutilation Act of 1995 [8]. Additionally, travel out of the country for FGM/C procedures, so called “vacation cutting,” became illegal in 2013 under the Transport for Female Genital Mutilation Act [9]. A 2017 case in Michigan of a US-licensed doctor performing FGM/C on nine girls illustrates the complexity of attitudes leading to controversial legal decisions and a challenge to the federal laws [10]. Currently, there are only 40 states with laws against FGM/C [11].

Despite the increasing pertinence of FGM/C risk to young children in western countries, there are few studies that focus on pediatricians, who are uniquely positioned to identify risk and prevent the practice [12]. Previous studies have highlighted that pediatricians in Australia and the US have little to no experience with FGM/C, are not trained to diagnose or manage FGM/C, and are not conducting routine external genital examinations on their female patients, such that FGM/C is often missed [13, 14].

Reductions in prevalence have been slow, prompting a need for more guidance and clinical support for the prevention of the practice. A 2008 international interagency—including the WHO and United Nations Children's Fund, among others—statement titled “Eliminating Female Genital Mutilation” called to uphold the rights of girls and women and take actions to end FGM/C [1]. Subsequently, multiple clinical groups, including the WHO and American Academy of Pediatrics (AAP), have released evidence-informed recommendations and practical handbooks for health care providers [4, 15]. The AAP Clinical Report is the first comprehensive summary of FGM/C in children [15]. These documents have emphasized the importance of easy access to educational opportunities and tools for pediatricians. However, there is currently no standardized national or international clinical practice guideline that focuses on pediatric FGM/C, and there is no required medical training for FGM/C in the US [12–17]. In addition, there are very few studies in the US and other countries that have focused on knowledge or actions addressing child protection strategies [12].

Our study aims to begin to fill this gap by surveying a national sample of US child abuse pediatricians (CAPs) with the objective of documenting and better understanding their knowledge, attitudes, and clinical practice regarding FGM/C. We hypothesize that CAPs that have received training about FGM/C will have increased knowledge about and ability to identify and manage FGM/C. Understanding the gaps in CAPs’ knowledge about FGM/C will help identify the training needs of those physicians who are in a position to serve as experts and educators for general pediatricians about these cases.

## Methods

### Participant recruitment

This study is a cross-sectional survey of CAPs with a focus on their attitudes, knowledge, clinical experience, and education surrounding FGM/C. Participants were recruited from the Ray E. Helfer Society, the primary subspecialty society for physicians devoted to the problem of maltreated children and seeking to provide medical leadership concerning child abuse and neglect. The Helfer Society counts among its members most board-certified CAPs in the US. At the time of this study, there were 604 members. Only US Helfer members were eligible—making 510 eligible for the study. At the time of the study, there were 232 board-certified CAPs in the US. As board certification is relatively new for child abuse pediatrics, there are physicians who practice as a CAP without board-certification and were included in this study.

Subjects were sent an email via the Helfer Society listserv between September 2020 and October 2020 with the link to the survey on SurveyMonkey. After an initial email, two additional follow up emails were sent at two week intervals. Respondents were included if they were members of the Helfer Society as assumed by their appearance on the email listserv, agreed to participate in the study as described in a consent statement before the survey began, were a physician practicing in the US, and completed any section of the domains described below. Respondents were not included if they did not meet these criteria (see Supplementary Material 1). The study was approved by the Columbia University Irving Medical Center Institutional Review Board.

### Survey development

We developed a survey based on previous studies and peer-reviewed by experts in the field [13, 14, 17, 18]. Adjustments were then made based on feedback from FGM/C experts from the European Network Care and Share against Female Genital Mutilation group and clinicians working with asylum clinics [19]. The final survey included 50 questions in five domains: clinician demographics, attitudes and awareness of FGM/C, clinical practice regarding FGM/C, knowledge of FGM/C, and training and education surrounding FGM/C (see Supplementary Material 1). The types of questions included were multiple choice and Likert scales that ranged from strongly agree to strongly disagree. Given the sensitive nature of this topic and the length of the survey, participants were not required to submit responses to every question. Subsequently, there are varying response rates to each question, which is specified within the results and range from an *n* of 65 to 46.

### Statistical analysis

Scores for knowledge of FGM/C were calculated based on correct responses to each question and added together for a composite score. Questions were designed to include a correct response for knowledge questions based on known statistics and facts about FGM/C. For example, a question that tested respondents’ knowledge of reportable cases with brief case vignettes based on federal laws. Associations between scores and demographic groups were assessed using Kruskal–Wallis tests. Associations between confidence and knowledge were assessed using Fisher’s exact test. P-values were considered significant at a threshold of 0.05; confidence intervals are reported for values found to be statistically significant. The analysis was conducted in R, an open source statistical programming language [20].

## Results

### Sample characteristics

Of the 604 email invitations, 510 members who practice in the US were eligible. Of those, 65 (12.7%) provided responses for at least one domain related to FGM/C. As discussed in the Methods section, the number of respondents varies with each question due to the length and sensitivity of the survey and subject. A majority (81%) of respondents were female, nearly half (47%) were over 50 years old, and most (72%) identified as White/Caucasian (Table 1). Most (89%) respondents identified child abuse pediatrics as their main area of practice, with the majority (69%) in practice for over 10 years. Over 75% practiced in an urban setting and 88% in an academic practice. The majority (72%) served a patient population that was less than 20% immigrant or refugees (Table 2).

**Table 1:** Baseline characteristics of respondents who completed at least one domain related to FGM/C (total *n* = 65)

Category	n (%)
**Gender**	
Female	52 (81.3%)
Male	12 (18.8%)
**Age**	
< 35 years	9 (14.1%)
35–49 years	25 (39.1%)
50–64 years	17 (26.6%)
> 65 years	13 (20.3%)
**Race/Ethnicity**	
White/Caucasian	46(71.8%)
Black/African American	5 7.8%)
Asian/Pacific Islander	3 (4.7%)
Latinx	4 (6.3%)
Multiple Ethnicity/Other	5 (7.8%)
**Religion**	
Christian	17 (26.6%)
No religion	14 (21.9%)
Catholic	12 (18.8%)
Judaism	8 (12.5%)
Protestant	7 (10.9%)
Other	2 (3.1%)
Prefer not to answer	4 (6.3%)

**Table 2:** Characteristics of the clinical practice of respondents who completed at least one domain related to FGM/C (total *n* = 65)

Category	n (%)
**Specialty Training**	
Child Abuse Pediatrics	38 (58.4%)
General Pediatrics	16 (24.6%)
Other	11 (16.9%)
**Length of Practice**	
< 5 years	13 (20.0%)
5–10 years	7 (10.8%)
> 10 years	45 (69.2%)
**Area of Practice**	
Child Abuse Pediatrics	58 (89.2%)
General Pediatrics	3 (4.6%)
Other	4 (6.2%)
**Weekly Pediatric Patient Volume**	
< 10	24 (38.7%)
10–19	17 (27.4%)
> 20	21 (33.9%)
**Region of Practice**	
Northeast	18 (27.7%)
Midwest	19 (29.2%)
South	20 (30.8%)
West	8 (12.3%)
**Practice Location**	
Urban	49 (75.4%)
Suburban	10 (15.4%)
Rural	4 (6.2%)
Other	2 (3.1%)
**Type of Practice**	
Academic	57 (87.7%)
Federally Qualified Health Center	3 (4.6%)
Other	6 (7.7%)
**Immigrant/Refugee Patient Population**	
0–20%	46 (71.9%)
21–40%	13 (20.3%)
41–60%	5 (7.8%)

### Attitudes and awareness

All 65 respondents had previously heard of FGM/C, and 45 (70%) believed they have an important role in preventing the practice. Almost all respondents agreed that all types of FGM/C are harmful (95%), are illegal in the United States (92%), are a violation of human rights (99%), and are a traditional cultural practice in some cultural groups (97%) (Table 3). Only 9 (14%) respondents believed that the practice is required by religion; however, 26 (40.0%) were not sure if the practice is required by religion. Most (74%) agreed that FGM/C is performed in children in the United States. Knowledge/awareness of FGM/C did not significantly vary by gender (*p* = 0.65), age (*p* = 0.11), race (*p* = 0.58), practice length (*p* = 0.15), region (*p* = 0.79), or refugee proportion of patient population (*p* = 0.75). A majority (63%) were able to correctly identify countries from a list as having high prevalence— >80% of reproductive age girls and women have undergone FGM/C [7].

**Table 3:** Child Abuse Pediatricians’ awareness, attitudes, and knowledge about FGM/C. (*n* = 65)

**Statement about attitudes and awareness**	**Agree, n (%)**
All types of FGM are harmful	62 (95.4%)
Performing any type of FGM/C is illegal in the USA	60 (92.3%)
FGM/C is a violation of human rights	64 (98.5%)
In some cultural groups, FGM/C is a traditional cultural practice	63 (96.9%)
The practice of FGM/C is required by religion	9 (13.8%)
FGM/C is performed in children in USA	48 (73.8%)
**Example of FGM/C**	**Correctly identify whether to report or not, n (%)**
Child cut abroad before immigrating to the US (not reportable)	38 (58.5%)
Planned vacation cutting (reportable)	52 (80.0%)
Perceived risk based on family culture (not reportable)	55 (84.6%)
Child who has had FGM/C performed in the US (reportable)	60 (92.3%)

### Knowledge about FGM/C policy and clinical guidelines

Of 54 respondents, 23 (43%) were aware of the Interagency Statement, “Eliminating Female Genital Mutilation.”

Nearly half (48%) of respondents were not aware of the WHO clinical handbook on the “Care of Girls and Women Living with Female Genital Mutilation.” Most (70%) were aware of the recently published AAP clinical report on FGM/C.

### Knowledge of laws and reporting guidelines

Of the 54 respondents, 34 (63%%) were not aware of federal laws mandating reporting of FGM/C, and 40 (74%) were not aware of their own state’s laws around FGM/C. Few had read the laws. Regarding clinical practice of reporting, of 64 respondents, 53 (83%) indicated that it would not be difficult for them to report a case of FGM/C to Child Protective Services (CPS). Common reasons for difficulty reporting included fear of damaging the patient-provider relationship, limited legal knowledge, concern that reporting could cause legal trouble for the family, and discomfort with passing judgment on a cultural practice.

To evaluate knowledge of reporting laws, four cases were posed to respondents (Table 3). Overall, most respondents would correctly report to CPS if a child had FGM/C performed in the US (92%) and if there was a case of planned vacation cutting (80%). Most (85%) would correctly not report a scenario where there was perceived risk based on family culture. Notably, only 38 (59%) would correctly not report a case of a child cut abroad before immigrating to the US, and 11 (17%) respondents indicated that they were unsure about whether to report the case. These findings did not significantly vary by gender (*p* = 0.27), age (*p* = 0.45), race (*p* = 0.48), practice length (*p* = 0.09), region (*p* = 0.79), and refugee proportion of patient population (*p* = 0.68).

### Knowledge and confidence of identifying WHO types and subtypes

**Fig. 2:**
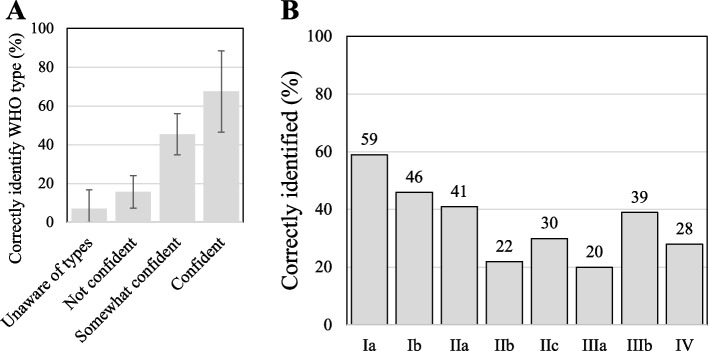
Child abuse pediatricians’ identification of WHO subtypes. (**A**) Correct identification of WHO types of FGM/C by level of confidence. Error bars represent + 1 standard error. (*n* = 46); (**B**) Correct identification of WHO types of FGM/C by subtype. (*n* = 46)

Of 54 respondents, 47 (87%) were aware of the WHO types and subtypes of FGM/C, but many were not confident in their ability to and were unable to accurately identify them. Only 5 (9%) felt confident distinguishing between the WHO FGM/C types and subtypes. Respondents were asked to identify the WHO types and subtypes with representative images (Fig. 1) [2]. Scores for correctly identified types differed based on self-determined confidence to identify WHO-types of FGM/C (*p* < 0.001) (Fig. 2). Individuals who were unaware of WHO types of FGM/C or were not confident in their ability to identify them scored significantly lower (median = 0/8 correct) than individuals who had some confidence or were confident that they could identify the types (median = 4/8 correct) (*p* < 0.001). Age (*p* = 0.31), gender (*p* = 0.73), race (*p* > 0.99), length of practice (*p* = 0.55), region (*p* = 0.33), and refugee proportion of patient population (*p* = 0.54) were not significantly associated with confidence. However, individuals reporting previous training related to FGM/C were significantly more likely to report being confident in identifying WHO types of FGM/C (OR: 4.82, 95% CI 1.25 to 21.7, *p* ≤ 0.05) and scored significantly higher in their identification of these types (*p* ≤ 0.05) (Fig. 2).

### Clinical practice regarding FGM/C

Of those who had heard of FGM/C (*n* = 65), 55 (85%) rarely or never ask about FGM/C in their clinical interviews with patients, though 52 (80%) reported always or very often looking for FGM/C in their physical exams. Only one (2%) has been asked for advice on where they could access FGM/C. No respondents had been asked to perform FGM/C.

Ten (15%) respondents had previously reported FGM/C to CPS whether or not they had made the diagnosis. Reasons for cases that had been reported included concern of perceived risk due to travel to a country with a high prevalence of FGM/C, confirmed planned vacation cutting, cases performed in the US, and cases of patients who were cut before immigrating to the US.

### Training and education about FGM/C

Only 11 (21%) of 53 respondents felt comfortable discussing FGM/C with parents from countries with a high prevalence of FGM/C. When asked, 35 (66%) reported having received prior training about FGM/C, including self-directed learning (49%), specialist training (34%), and a formal course that included FGM/C (23%). With regard to educational materials, respondents were most interested in a guide to recognize and classify FGM/C (60%), a guide to laws about FGM/C (59%), information/resources for patients/parents (55%), and a guide to asking patients about FGM/C (47%).

## Discussion

This is the first national survey of US CAPs regarding FGM/C. CAPs who had received prior training were both more confident at and more accurate at identifying WHO types and subtypes of FGM/C. While CAPs demonstrated high rates of training related to FGM/C, respondents identified a need for further training related to cultural competency to manage FGM/C. Additionally, most CAPs demonstrate a limited knowledge and awareness of reporting policies. Laws about FGM/C are nuanced and vary by state, which may lead to underreporting or potentially inappropriate reporting.

## General Knowledge

A majority (66%) of CAPs from our study indicated that they had previous training related to FGM/C compared to only 28% of US general pediatricians [14] and 14.5% of Australian general pediatricians [13]. This higher rate of training is expected given that CAPs specialize in child abuse. Accordingly, US CAPs consistently look for FGM/C during their examinations at a much higher rate than Australian general pediatricians, which may reflect CAP’s specialized training to identify trauma [13].

However, despite higher rates of training related to FGM/C, CAPs had similar rates of common misconceptions about FGM/C, such as the belief that it is required by religion, compared to Australian general pediatricians. A majority of respondents were also unaware of FGM/C related resources. And, despite often looking for FGM/C, few feel comfortable discussing FGM/C with parents indicating that FGM/C remains a sensitive topic to ask about. Forming a trusting relationship to ask sensitive questions about FGM/C may be facilitated by establishing proper, quality communication in the setting of linguistic barriers, having an in-community facilitator, and avoiding dichotomous statements to move towards bias-free interactions [15, 21].

These findings highlight the importance of raising awareness of guidelines through promotion by major medical organizing bodies, such as the recently released AAP clinical report and the recently released diagnostic illustrated guide [15, 22]. US CAPs identified a desire for more education about how to ask at-risk families about FGM/C suggesting that training related to cultural competency about FGM/C is an important gap to fill for physicians’ ability to approach the topic. Therefore, a specialist trained in FGM/C should always be consulted if the physical findings are equivocal and risk factors for FGM/C are present [15].

## Confidence and Ability of Identification of WHO Types and Subtypes

Most US CAPs (87%) were aware of the WHO types and subtypes compared to only 22% of Australian general pediatricians, highlighting the benefits of specific CAP training about FGM/C [13]. However, most US CAPs (91%) do not feel confident identifying different types of FGM/C at a similar proportion (89%) to US general pediatricians despite more US CAPs having training in FGM/C and genital exams than general pediatricians [14].

Creighton and Hodes suggest that CAPs who have extensive training in genital examination tend to concentrate on hymenal and anal findings and may not examine the clitoris in detail unless looking specifically for FGM/C, which may contribute to their lack of confidence identifying FGM/C [23]. Additionally, less mutilating types of FGM/C, such as type IV, which present with fewer complications and can result in inconclusive diagnosis, are becoming more prevalent, emphasizing the importance of consulting an expert on these cases [24–26].

Importantly, we found that individuals were able to accurately gauge their ability to identify WHO-types of FGM/C, and previous training was associated with more confidence and significantly more accurate identification of types of FGM/C. This suggests the possibility that training related to FGM/C may increase both confidence and ability to identify FGM/C [27].

## Reporting Policy Knowledge

Previous studies have not addressed reporting policies and practices of pediatricians regarding FGM/C. One study surveyed French general practitioners and found that less than a quarter of respondents correctly identified cases or responded to questions related to judicial reporting procedures in France [18]. Overall, most US CAPs do not find it difficult to report cases of FGM/C, which is consistent with their specialty training. However, like French general practitioners, those US CAPs who did find it difficult to report cases of FGM/C cited stigma, deteriorating the patient-provider relationship, and complexity of child abuse reporting procedure [18].

Our findings of CAPs reporting knowledge support an unfamiliarity with some more nuanced reporting policies. Most US CAPs were able to correctly identify reportable and non-reportable scenarios of FGM/C, indicating that CAPs are aware of reporting mandates. However, most were not aware of federal or state laws about FGM/C and mistakenly reported cases where a child was cut before immigrating to the US. This suggests that some reporting mandates may be missed.

## Limitations

There are several limitations to this study. Our response rate (12.7%) is low when compared to similar survey-based studies of FGM/C and to Helfer Society surveys. Additionally, 18 eligible respondents did not complete the survey such that the sample size was too small to draw generalizable conclusions. Response rates also varied by question further diminishing the sample size for certain portions of the survey. The length of the survey as indicated in the questionnaire feedback may have contributed to this. While this survey was developed in conjunction with experts in the field, it was not piloted with non-experts or a sample of CAPs, which may have preemptively revealed issues with the survey that contributed to the low response rate. As with many surveys, we assume that those who have an interest in FGM/C may respond at higher rates and result in selection bias and an overestimate of national FGM/C knowledge. Given the low response rates, we emphasize caution in interpretation of results as a complete representation of the current knowledge of CAPs.

In addition, as indicated in the questionnaire feedback, three respondents expressed concern regarding the quality and size of some images. This may have contributed to both the completion of the survey and inaccurately interpreting the images. Finally, respondents’ ability to recognize types by image may or may not correlate with their competence in diagnosing and reporting FGM/C in a clinical setting.

## Conclusions

This study demonstrates that while US CAPs have a high rate of training related to FGM/C, they have limited knowledge of FGM/C in general highlighting the importance of access to guidelines like the recent AAP Clinical Report and illustrated guide to FGM/C [15, 22]. CAPs are highly trained in discussion about sensitive topics; however, we find that FGM/C remains a topic that CAPs find difficult to discuss with families. CAPs are uniquely positioned to serve as educators for general pediatricians to aid in diagnosing and correctly reporting FGM/C. CAPs have the opportunity to be the experts about how best to approach families in a respectful, culturally sensitive, and non-stigmatizing fashion to help manage and prevent the practice. In order to better equip CAPs for the potential leadership role in addressing and preventing FGM/C, a future qualitative or mixed methods study of US CAPs that further improves our understanding of the need for greater awareness of published guidelines and for optimal training methods is necessary.

### Supplementary Information

**Supplementary Material 1. **

**Supplementary Material 2. **

## Data Availability

The datasets used and/or analyzed during the current study are available from the corresponding author on reasonable request.

## Abbreviations

AAP: American Academy of Pediatrics
CAP: Child Abuse Pediatrician
CPS: Child Protective Services
FGM/C: Female Genital Mutilation/Cutting
US: United States
WHO: World Health Organization
