# Supplementary material for: Female genital mutilation and cutting: a survey of child abuse pediatricians
Source: BMC Womens Health. 2024 Jun 17;24:348. doi: 10.1186/s12905-024-03119-7 (PMC11181596; doi:10.1186/s12905-024-03119-7)
Supplement: Supplementary file 1 — Supplementary Material 1. [file 12905_2024_3119_MOESM1_ESM.pdf]

Supplementary Material 1. FGMC Supplementary Material 1 (Survey). [pdf format] Title: FGM/C Survey. This file includes all questions included in the survey utilized to collect data for this study.

FGM/C Survey:

Female Genital Mutilation and Cutting: A Survey of Child Abuse Pediatricians

Introduction Statement

Thank you for your interest in participating in this study. This anonymous survey takes 10-15 minutes to complete.

Study Description
This study's purpose is to better understand US pediatricians' knowledge, attitudes, clinical practices, and education surrounding female genital mutilation and cutting (FGM/C). The survey has 6 sections each represented as a separate page: Demographics, Attitudes, Practice, Knowledge, Training, and Other.

Risks and Benefits
This study will ultimately inform the development of FGM/C related training and educational resources that will enable pediatricians to provide better care to patients affected by FGM/C. We would like to address that this survey is focused on female genital mutilation and cutting (FGM/C), which may be a sensitive subject to some. If this is you, please take care of yourself and make an informed decision to or not to participate.

Voluntary Participation
Participating in this study is your choice. You may choose to participate, or you may refuse to participate. If you choose to begin the survey, you may change your mind at any point of the survey. You will not be paid or compensated otherwise for participating in this study.

Privacy Statement
All responses will be collected anonymously. No identifying information including names, email address, or IP addresses will be collected. Only researchers directly involved in this study will have access to the data that might identify you. Please note that the survey is being conducted with the help of Survey Monkey, a company not affiliated with Columbia and with its own privacy and security policies that you can find at its website. However, we anticipate that your participation in this survey presents no greater risk than everyday use of the internet.

Questions
If you have any questions about the research, please feel free to reach out to the research team at jb58@cumc.columbia.edu.

Columbia University IRB# AAAT1776

* 1. By clicking the response "I agree," I agree to participate in this research study, as described above:

- I agree

* 2. Are you a physician who practices in the United States?

- Yes, I practice in the United States
- No, I DO NOT practice in the United States.

Demographics of Clinician

1. What is your gender?
    - Female
    - Male
    - Non-Binary/Non-Conforming
    - Transman/Transmasculine/FTM
    - Transwoman/Transfeminine/MFM
    - Decline to answer

2. What was your age at your last birthday?
    - Less than 35 yrs
    - 35-49 yrs
    - 50-64 yrs
    - 65 yrs or greater

3. Which race/ethnicity best describes you? (Please choose only one.)
    - American Indian or Alaskan Native
    - Asian/Pacific Islander
    - Black or African American
    - Hispanic
    - Latino
    - White/Caucasian
    - Multiple Ethnicity/Other (please specify)

4. Do you identify with any of the following religions? (Please select all that apply.)
    - Buddhism
    - Catholic
    - Christian
    - Hindu
    - Islam
    - Judaism
    - Protestant
    - Prefer not to answer
    - No religion
    - Other (please specify)

5. How long have you been in practice?
- < 5 yr experience
- 5-10 yr experience
- > 10 yr experience

6. Which area of pediatrics best describes your practice?
- General Pediatrics
- Adolescent Medicine
- Child Abuse Pediatrics
- Other (please specify)

7. How did you receive this survey?
- AMA Listserv
- Helfer Society Listserv
- SAHM Listserv
- Other

8. Which of the following best describes the location of your primary clinical practice site?
- Urban
- Suburban
- Rural

9. Which of the following best describes the location of your primary clinical practice site?
- Private Practice -- Hospital
- Private Practice -- Clinic
- Academic -- Hospital
- Academic -- Clinic
- FQHC/CHC

10. Which state do you practice in? (Please input your state's two-letter abbreviation, e.g. NY)

11. What is the zip code of your practice? #####

12. In a typical week, how many children do you see who are under 18 yrs of age?

____

Attitudes & Awareness

13. Have you previously heard about FGM/C?
- Yes
- No

14. Please indicate your level of agreement with each of the following statements (select ONE box that applies to each statement):

	Strongly agree	Tend to agree	Not sure	Tend to disagree	Strongly disagree
All types of FGM/C are harmful					
Performing any type of FGM/C is illegal in the USA					
FGM/C is a violation of human rights					
In some cultural groups, FGM/C is a traditional cultural practice					
The practice of FGM/C is required by religion					
FGM/C is performed in children in USA					
You see a girl whose mother is Sudanese. The girl has a UTI and the mother tells you that they had FGM/C performed on the girl in the US a week ago. Notification to child protection services is mandatory.					
You see a US born girl whose mother is from Somalia. Notification to child protection services of this child's increased risk for FGM/C is mandatory.					
A mother of a girl tells you that the family intends to fly home next week to Yemen and have the girl cut there. Notification to child protection service is mandatory.					
You are seeing an 8 year old girl who was born in a Kenyan refugee camp to Somali parents. She arrived in the US 3 years ago and needs a proof of					

physical exam for school entry. You perform a full physical exam, including visualization of her external genitalia and she has type IIIa FGM/C. Records from prior well child care visits note that her external GU exam was deferred at each visit. Her mother confirms that the girl was cut before coming to the US. Notification to child protection services is mandatory.					

15. In the USA, FGM/C is most likely to be practiced by individuals from the following countries (please select all options that apply):

- Egypt
- Eritrea
- Columbia
- Guinea
- India
- Indonesia
- Iraq
- Niger
- Sierra Leone
- Somalia
- Sudan
- Uganda
- Other (please specify): ___________________
- Don't know

16. Which of these religious groups may practice FGM/C? (Please select all that apply.)

- Christianity
- Islam
- Judaism
- Traditional Religion (e.g. indigenous religious beliefs and practices)
- Other (please specify)
- Don't know

Practice

17. What proportion of your patient population are considered immigrants or refugees?

- 0-20%
- 21-40%
- 41-60%

- 61-80%
- 81-100%

18. How often do you ask about FGM/C when taking a medical history?
    - Never
    - Rarely
    - Sometimes
    - Very often
    - Always

19. How often do you look for FGM/C during clinical genital examinations?
    - Never
    - Rarely
    - Sometimes
    - Very often
    - Always

For following questions 20-24, please respond considering only your clinical practice excluding during your training.

20. Have you ever diagnosed a child (0-18 years) who had undergone an FGM/C procedure?
    - Yes
    - No
    - Don't Know

If yes, how many cases have you seen? __________

21. Have you ever been approached by anyone for advice about where to have FGM/C done?
    - Yes
    - No

22. Have you ever been approached by anyone to perform FGM/C?
    - Yes
    - No

23. Have you ever reported a case of FGM/C?
    - Yes
    - No
    - Don't know

24. If you have made a report of FGM/C to Child Protective Services,

    ...was the FGM/C procedure done in the US?
    - Yes
    - No
    - Don't know

...were you informed by the family that the child was traveling abroad to have the procedure done outside of the US?

- Yes
- No
- Don't know

- Yes
- No
- Don't know

...was the child already cut abroad before immigrating to the US?

- Yes
- No
- Don't know

25. Would it be difficult for you to make a child abuse report if you came across a case of FGM/C?

- Yes
- No
- Don't know

26. If you think that you would have difficulty with child abuse reporting procedures for FGM/C, what would be the main difficulty you would have? (choose ONLY one answer)

- I do not think it is necessary to report because the procedure was already completed abroad before the patient arrived in the US.
- I am afraid of damaging the relationship with the family.
- I am concerned that reporting will not help the family.
- I don't know enough about FGM/C reporting laws.
- I do not have the time to make the call.
- I find FGM/C hard to judge because it is a traditional practice.
- I do not want to cause legal problems for my patient and the family.
- I am not sure how to make a report.
- I don't have any difficulties reporting because FGM/C is a mandatory report in the US.

27. Do you think that you have an important role in prevention of FGM/C?

- Yes
- No
- Don't know

Knowledge on FGM/C

28. Please indicate whether you are aware of, have read or have used the resources listed below (please select all options that apply):

Resource	I am not aware of this	I am aware of this	I have read this	I have read and used this in my practice
Eliminating Female Genital Mutilation: An Interagency Statement -- OHCHR, UNAIDS, UNDP, UNECA, UNESCO, UNFPA UNHCR, UNICEF, UNIFEM, WHO (2008)[1]				
World Health Organization -- Care of Girls and Women Living with Female Genital Mutilation. *A Clinical Handbook.* (2018)[2]				
World Health Organization -- Guidelines on the Management of Health Complications from FGM (2016)[3]				
UNICEF -- FGM/C: A Statistical Overview and Exploration of the Dynamics of Change (2013)[4]				
American Academy of Pediatrics Clinical Report -- Diagnosis, Management, and Treatment of FGM/C in Girls (2020)				

[1] World Health Organization. Eliminating female genital mutilation: an interagency statement-OHCHR, UNAIDS, UNDP, UNECA, UNESCO, UNFPA, UNHCR, UNICEF, UNIFEM, EHO. Geneva; 2008. Available at: http://apps.who.int/iris/bitstream/10665/43839/1/9789241596442_eng.pdf
[2] World Health Organization. Care of Girls and Women Living with Female Genital Mutilation. A Clinical Handbook. 2018. Available at: https://www.who.int/reproductivehealth/publications/health-care-girls-women-living-with-FGM/en/
[3] World Health Organization. Guidelines on the Management of Health Complications from Female Genital Mutilation. 2016. Available at: https://www.who.int/reproductivehealth/topics/fgm/management-health-complications-fgm/en/.
[4] UNICEF. Female genital mutilation/cutting: A statistical overview and exploration of dynamics of change. 2013. Available at: http://data.unicef.org/resources/female-genital-mutilationcutting-statistical-overview-exploration-dynamics-change/

American Academy of Pediatrics -- Immigrant Toolkit[5]				
Centers for Disease Control and Prevention -- Domestic Refugee Screening Guidelines[6]				
FGM/C, US Citizenship and Immigration Services background information and educational pamphlets [7]				
FGM: A Visual Reference and Learning Tool for Health Care Professionals. *Video.* (Abdulcadir et al, 2016)[8]				
U.S. Federal Legislation on FGM/C [9]				
Your own State's Legislation on FGM/C				
Please specify any other resources on FGM that you are aware of but not listed above: ***				

29. How confident are you in distinguishing between the four WHO FGM/C types and their subtypes?

- Confident
- Somewhat confident
- Not confident
- I am not aware of the different types of FGM/C

[5] American Academy of Pediatrics. Immigrant Toolkit. Available at: https://www.aap.org/en-us/Documents/cocp_toolkit_full.pdf
[6] Centers for Disease Control and Prevention. Domestic Refugee Screening Guidelines. Available at: http://www.cdc.gov/immigrantrefugeehealth/guidelines/domestic/domesticguidelines/html
[7] Female Genital Mutilation or Cutting (FGM/C), US Citizenship and Immigration Services background information and educational pamphlets in Amharic, Arabic, French, Somali, Swahili, and Tigrinya. Available at: http://www.uscis.gov/fgmc
[8] Abdulcadir J, Marras S, Catania L, Hindin MJ, Say L, Petignat P, Abdulcadir O. Femail Genital Mutilation. A Visual Reference and Learning Tool for Health Care Professionals. *Obstet Gynecol.* 2016; 128:958-963. Video available at: https://www.youtube.com/watch?v=XRid7jIUzMY
[9] "The Federal Prohibition of Female Genital Mutilation of 1995" and 2013 amendment -- 18 U.S. Code § 116.Female genital mutilation

Citation for the following diagram-based questions: Abdulcadir, Jasmine MD; Catania, Lucrezia MD; Hindin, Michelle Jane PhD; Say, Lale MD; Petignat, Patrick MD; Abdulcadir, Omar MD Female Genital Mutilation, Obstetrics & Gynecology: November 2016 - Volume 128 - Issue 5 - p 958-963 doi: 10.1097/AOG.0000000000001686

30. Please select the image that shows the WHO Type Ia classification of FGM/C: (Pink indicates part of genitalia DEFINITELY affected; Orange indicates parts of genitalia that MAY be affected)

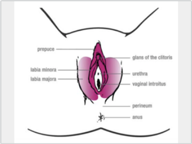
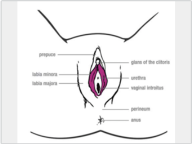
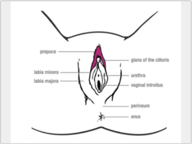
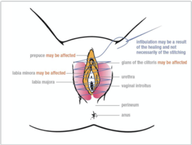
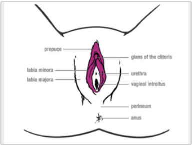
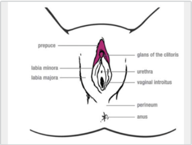
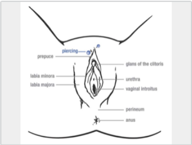
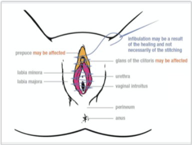

Don't know

31. Please select the image that shows the WHO Type Ib classification of FGM/C: (Pink indicates part of genitalia DEFINITELY affected; Orange indicates parts of genitalia that MAY be affected)

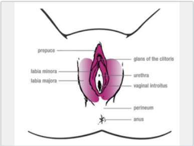
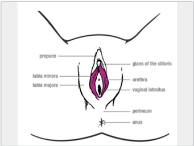
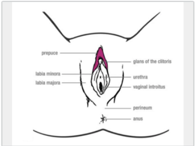
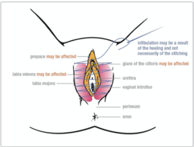
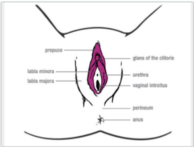
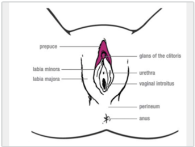
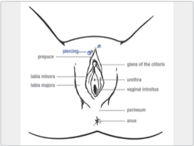
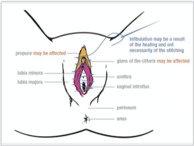

Don't know

32. Please select the image that shows the WHO Type IIa classification of FGM/C: (Pink indicates part of genitalia DEFINITELY affected; Orange indicates parts of genitalia that MAY be affected)

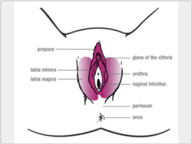
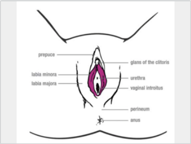
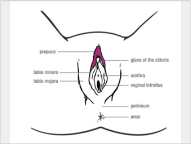
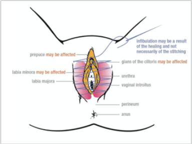
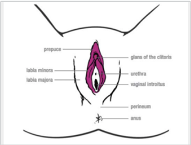
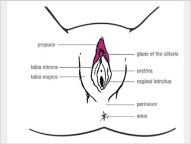
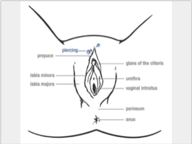
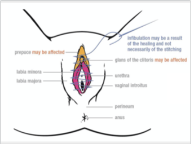

Don't know

33. Please select the image that shows the WHO Type IIb classification of FGM/C: (Pink indicates part of genitalia DEFINITELY affected; Orange indicates parts of genitalia that MAY be affected)

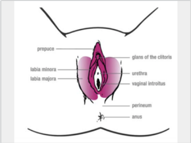
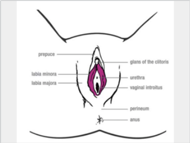
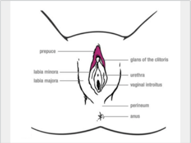
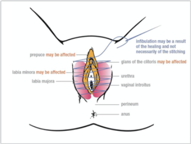
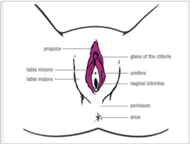
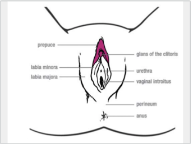
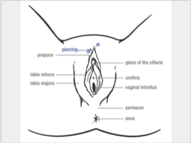
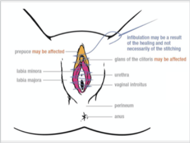

Don't know

34. Please select the image that shows the WHO Type IIc classification of FGM/C: (Pink indicates part of genitalia DEFINITELY affected; Orange indicates parts of genitalia that MAY be affected)

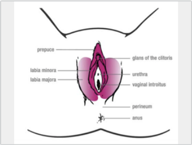
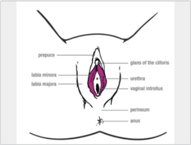
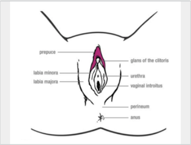
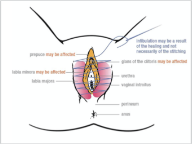
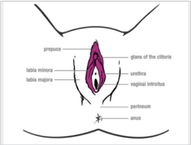
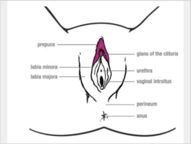
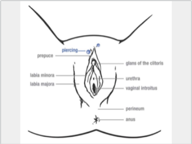
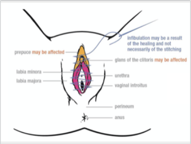

Don't know

35. Please select the image that shows the WHO Type IIIa classification of FGM/C: (Pink indicates part of genitalia DEFINITELY affected; Orange indicates parts of genitalia that MAY be affected)

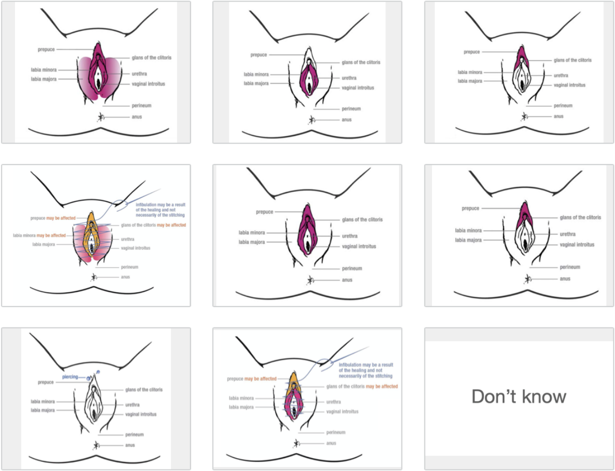

36. Please select the image that shows the WHO Type IIIb classification of FGM/C: (Pink indicates part of genitalia DEFINITELY affected; Orange indicates parts of genitalia that MAY be affected)

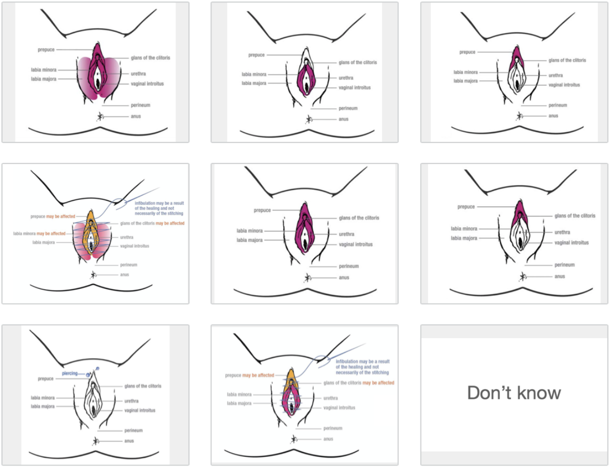

37. Please select the image that shows the WHO Type IV classification of FGM/C: (Pink indicates part of genitalia DEFINITELY affected; Orange indicates parts of genitalia that MAY be affected)

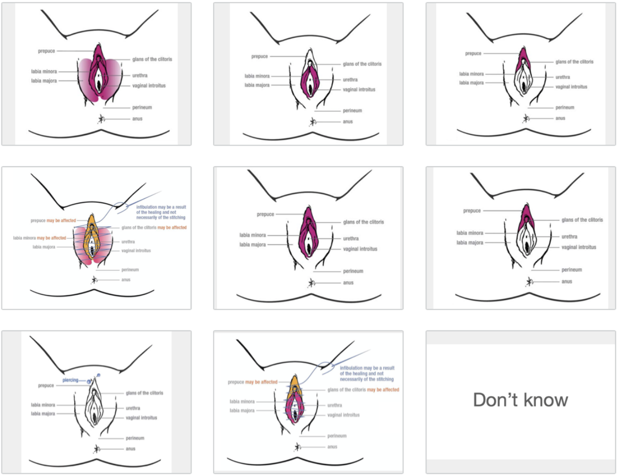

38. Are you aware of the short-term complications of FGM/C?
- Yes
- No

If yes, please specify: ___________________________________

39. Do you believe there are long-term consequences of FGM/C for health and well-being?
- Yes
- No
- Don’t know

If yes, please specify: ___________________________________

Training and Education

40. How comfortable are you discussing FGM/C with parents from countries with a high prevalence of FGM/C?
- Comfortable
- Somewhat comfortable
- Not comfortable
- I don’t discuss/address this issue in my practice

41. Have you ever had any education or training about FGM/C?
- Yes
- No

42. *If Yes*, please select the appropriate box:
    - ○ During undergraduate medical training
    - ○ Part of continuing or post-graduate medical education
    - ○ Self-directed learning activities
    - ○ Specialist training
    - ○ Specific courses (e.g. child protection that include FGM/C)

42. Would you like to receive educational materials for yourself?
- Yes
- No

43. Would you like to receive educational materials for patients?
- Yes
- No

44. What kinds of educational resources would help you most in dealing with FGM/C? (please select all options that apply):
- Information resources for patients/parents, eg. fact sheets
- A guide to asking patients about FGM/C

- A guide to genital examination for health professionals
- A guide to recognition and classification of FGM/C in children and young people
- Outline of US federal law and state specific laws/procedures and obligations of health professionals
- Information about the cultural context of FGM/C
- A guide for referral of patients with FGM/C
- Other(specify): _______________________________
- None

45. Would you be interested in an evidence-based educational module for FGM/C?
    - Yes
    - No

Other

46. Do you think that COVID-19 will affect applications for asylum in the US?
    - Yes
    - No
    - Don't know

47. Do you have any comments you would like to make?

____
