# Supplementary material for: Female genital mutilation and cutting: a survey of child abuse pediatricians
Source: BMC Womens Health. 2024 Jun 17;24:348. doi: 10.1186/s12905-024-03119-7 (PMC11181596; doi:10.1186/s12905-024-03119-7)
Supplement: Supplementary file 2 — Supplementary Material 2. [file 12905_2024_3119_MOESM2_ESM.pdf]

Supplementary Material 2. FGMC Supplementary Material 2 (Vignette Justifications). [pdf format] Title: Brief Clinical Vignette Justifications. This files include justifications for the clinical vignettes used in the survey to test knowledge for when to report FGMC cases.

Brief Clinical Vignette Justifications

Female Genital Mutilation and Cutting: Knowledge and Attitudes of Pediatricians in the United States

Scenario	Answer	Justification
You see a girl whose mother is Sudanese. The girl has a UTI and the mother tells you that they had FGM/C performed on the girl in the US a week ago. Notification to child protection services is mandatory.	YES	This scenario indicates that the child has had FGM/C in the US, which is illegal per Federal Prohibition of Female Genital Mutilation Act of 1995. This is reportable of both the parents and the person who performed the procedure.
You see a US born girl whose mother is from Somalia. Notification to child protection services of this child's increased risk for FGM/C is mandatory.	NO	This scenario exemplifies how simply belonging to a family or culture that practices FGM/C alone does not necessarily put the child at risk of FGM/C. Therefore, this scenario is not reportable in the US. This is in contrast to the UK where it is reportable, and both the child and mother would have to be examined.
A mother of a girl tells you that the family intends to fly home next week to Yemen and have the girl cut there. Notification to child protection service is mandatory.	YES	Vacation cutting is illegal in the US per the federal 2013 Transportation for Female Genital Mutilation law. Therefore, this is an example of risk that is reportable, because there is a plan in place for travel with intent to have the procedure performed and an imminent threat of FGM/C that can be prevented by reporting it.
You are seeing an 8-year-old girl who was born in a Kenyan refugee camp to Somali parents. She arrived in the US 3 years ago and needs a proof of physical exam for school entry. You perform a full physical exam, including visualization of her external genitalia and she has type IIIa FGM/C. Records from prior well childcare visits note that her external GU exam was deferred at each visit. Her mother confirms that the girl was cut before coming to the US. Notification to child protection services is mandatory.	NO	This scenario includes a girl who was cut abroad prior to arrival to the US. She had prior routine genital exam, however, often genital exams are marked as normal GU exam because physicians are only looking for Tanner stages of pubic hair and don't visualize anatomic structure. The exam in this scenario revealed Type IIIa done BEFORE immigrating to and arriving in the US.<br><br>If the mother confirms cutting of her daughter occurred prior to US arrival, there is no need to report. It is important to assure that the child has no medical complications and that if other daughters are US born, that the mother understands that it is illegal to send the child back to the country of origin for circumcision.
